# The Pathogenic Mechanism of the *Mycobacterium ulcerans* Virulence Factor, Mycolactone, Depends on Blockade of Protein Translocation into the ER

**DOI:** 10.1371/journal.ppat.1004061

**Published:** 2014-04-03

**Authors:** Belinda S. Hall, Kirsti Hill, Michael McKenna, Joy Ogbechi, Stephen High, Anne E. Willis, Rachel E. Simmonds

**Affiliations:** 1 Department of Microbial and Cellular Sciences, Faculty of Health and Medical Sciences, University of Surrey, Guildford, United Kingdom; 2 The Babraham Institute, Babraham, Cambridge, United Kingdom; 3 Faculty of Life Sciences, University of Manchester, Manchester, United Kingdom; 4 MRC Toxicology Unit, Leicester, United Kingdom; University of New Mexico, United States of America

## Abstract

Infection with *Mycobacterium ulcerans* is characterised by tissue necrosis and immunosuppression due to mycolactone, the necessary and sufficient virulence factor for Buruli ulcer disease pathology. Many of its effects are known to involve down-regulation of specific proteins implicated in important cellular processes, such as immune responses and cell adhesion. We have previously shown mycolactone completely blocks the production of LPS-dependent proinflammatory mediators post-transcriptionally. Using polysome profiling we now demonstrate conclusively that mycolactone does not prevent translation of TNF, IL-6 and Cox-2 mRNAs in macrophages. Instead, it inhibits the production of these, along with nearly all other (induced and constitutive) proteins that transit through the ER. This is due to a blockade of protein translocation and subsequent degradation of aberrantly located protein. Several lines of evidence support this transformative explanation of mycolactone function. First, cellular TNF and Cox-2 can be once more detected if the action of the 26S proteasome is inhibited concurrently. Second, restored protein is found in the cytosol, indicating an inability to translocate. Third, *in vitro* translation assays show mycolactone prevents the translocation of TNF and other proteins into the ER. This is specific as the insertion of tail-anchored proteins into the ER is unaffected showing that the ER remains structurally intact. Fourth, metabolic labelling reveals a near-complete loss of glycosylated and secreted proteins from treated cells, whereas cytosolic proteins are unaffected. Notably, the profound lack of glycosylated and secreted protein production is apparent in a range of different disease-relevant cell types. These studies provide a new mechanism underlying mycolactone's observed pathological activities both *in vitro* and *in vivo*. Mycolactone-dependent inhibition of protein translocation into the ER not only explains the deficit of innate cytokines, but also the loss of membrane receptors, adhesion molecules and T-cell cytokines that drive the aetiology of Buruli ulcer.

## Author Summary

Buruli ulcer is a progressive necrotic skin lesion caused by infection with the human pathogen *Mycobacterium ulcerans*. Mycolactone, a small compound produced by the mycobacterium, is the root cause of the disease pathology, but until now there has been no unifying mechanism explaining why. We have been using a model system to investigate the reason for the selective loss of protein that is a common feature of mycolactone exposure. Specifically, this involves identifying the point at which it stops immune cells making inflammatory mediators. In this work, we demonstrate that mycolactone inhibits production of such proteins by blocking the first step of protein export: translocation into a cellular compartment called the endoplasmic reticulum (ER). Proteins due for export are instead made in the cell cytosol where they are recognised as being in the wrong place and are rapidly degraded, causing a general cessation of the production of proteins that have to travel through the ER, including almost all secreted and surface proteins. This has a profound effect on basic cell functions such as growth, adhesion and survival. Therefore, we have identified the molecular basis underlying the key features of Buruli ulcer, and this will transform our understanding of disease progression.

## Introduction

Mycolactone is a lipid-like polyketide macrolide virulence factor produced by *Mycobacterium ulcerans*, the infectious agent of Buruli ulcer (BU) [1], [2]. This progressive, necrotizing, cutaneous lesion is common in West Africa but also found in other regions, including Australia, Asia and South America. Mycolactone is a key factor in BU pathology: possession of a plasmid carrying enzymes involved in mycolactone synthesis is essential for virulence and injection of mycolactone alone can reproduce many characteristics of the infection, including ulceration, necrosis and suppression of immune responses [1], [3]. Mycolactone has been shown to have diverse effects on a range of cells and tissues but a unifying mechanism underlying its pleiotropic actions has remained elusive.

*In vitro*, exposure to pure mycolactone is cytotoxic for many cell lines, but the dose and exposure required is highly variable ([4] and references therein) and primary immune cells (including T-cells, monocytes and macrophages) are considerably more resistant [5], [6]. While early evidence from cell lines implicated G1/G0 growth arrest and apoptosis [7], recent work showed that a more likely mechanism driving cell death *in vivo* is anoikis due to direct binding of mycolactone to the Wiskott-Aldrich Syndrome Protein (WASP), leading to inappropriate activation of WASP and relocalisation of the actin nucleating complex Arp2/3 [8]. This disrupts the cytoskeleton, altering cell adhesion and migration. Detachment of monolayer cells is a common feature of the mycolactone response and precedes cell death by up to 48 hours.

One of the most striking characteristics of BU lesions is an almost complete absence of inflammation despite extensive tissue damage. In ulcerated lesions, where large amounts of mycolactone are produced by foci of extracellular bacilli, inflammatory cell infiltration is limited to the periphery [9]–[11]. Infection is accompanied by alterations in local and systemic immune responses in which mycolactone plays a central role [11]–[14], via direct and indirect effects on T-cells, dendritic cells, monocytes and macrophages [5], [15]–[17]. Mycolactone interferes with T-cell activation, down-regulating expression of the T-cell receptor and reducing IL-2 production in response to activating signals [15], [17], [18]. Lymphocyte homing is also impaired due to suppression of L-selectin and LFA-1 levels, leading to a dramatic depletion of T-cells in peripheral lymph nodes [6]. In monocyte-derived dendritic cells, mycolactone inhibits the production of costimulatory molecules (such as CD40 and CD86). In addition, secretion of various cytokines and chemokines is blocked and mycolactone treated dendritic cells show a reduced ability to activate T-cells [16].

The innate immunity provided by monocytes and macrophages is also suppressed by mycolactone. Tissue resident macrophages normally play a central role in mycobacterial infections. However, *M. ulcerans* differs from other pathogenic mycobacteria in that, except in very early infection, the vast majority of bacilli are not found within the host macrophage but are located extracellularly. Mycolactone inhibits key macrophage responses such as nitric oxide production and phagocytosis as well as phagosome maturation and acidification [2], [4], [19]. In addition, mycolactone prevents the induction of many proteins essential for driving inflammation, including TNF, other cytokines/chemokines (for example, IL-6, IL-8 and IP-10), and further inflammatory mediators (such as the prostaglandin synthetase Cox-2) [5], [10], [15].

There is good evidence that mycolactone diffuses through the lesion in advance of the proliferating bacilli and the necrotic centre (see for example [20]). Therefore, understanding exactly how this compound mediates its diverse immunosuppressive and cytotoxic effects on cells surrounding the developing lesion is crucial. As outlined above, many of these effects involve loss of expression of specific proteins, both induced and constitutive, such as inflammatory mediators. Consequently, the same molecular mechanism that prevents inflammatory protein production in the macrophage may also explain the inadequate protein production more generally. This makes it an excellent model system with which to examine the basic cell biology of mycolactone function, since the response is inducible by nature and it is therefore straightforward to separate new protein synthesis from baseline levels.

We have previously shown that inducible inflammatory mediator production is inhibited by a post-transcriptional mechanism, since mycolactone does not modulate the LPS-dependent activation of ERK, JNK, p38 MAPK or NFκB and induced levels of mRNA are maintained or even enhanced [5]. However there is no significant decrease in total protein synthesis, nor are phosphorylation patterns of Akt, p70S6K, eIF4E and eIF2α changed; a finding confirmed in another model system, Jurkat T-cells [17]. In the current manuscript we demonstrate conclusively that mycolactone does not selectively inhibit translation as predicted [2], [5], and instead blocks co-translational translocation into the ER. This leads to the rapid degradation of mislocalised proteins in the cytosol and hence loss of detectable expression. We show that the production of nearly all new glycosylated and secreted proteins ceases following mycolactone exposure, not only in macrophages but in fibroblasts, epithelial and endothelial cells. This mechanism therefore provides the necessary explanation for many of the pleiotropic effects of this unique molecule and accounts for much of the underlying disease pathology.

## Results

### Mycolactone does not inhibit the translation of proinflammatory mRNAs

**Figure 1:**
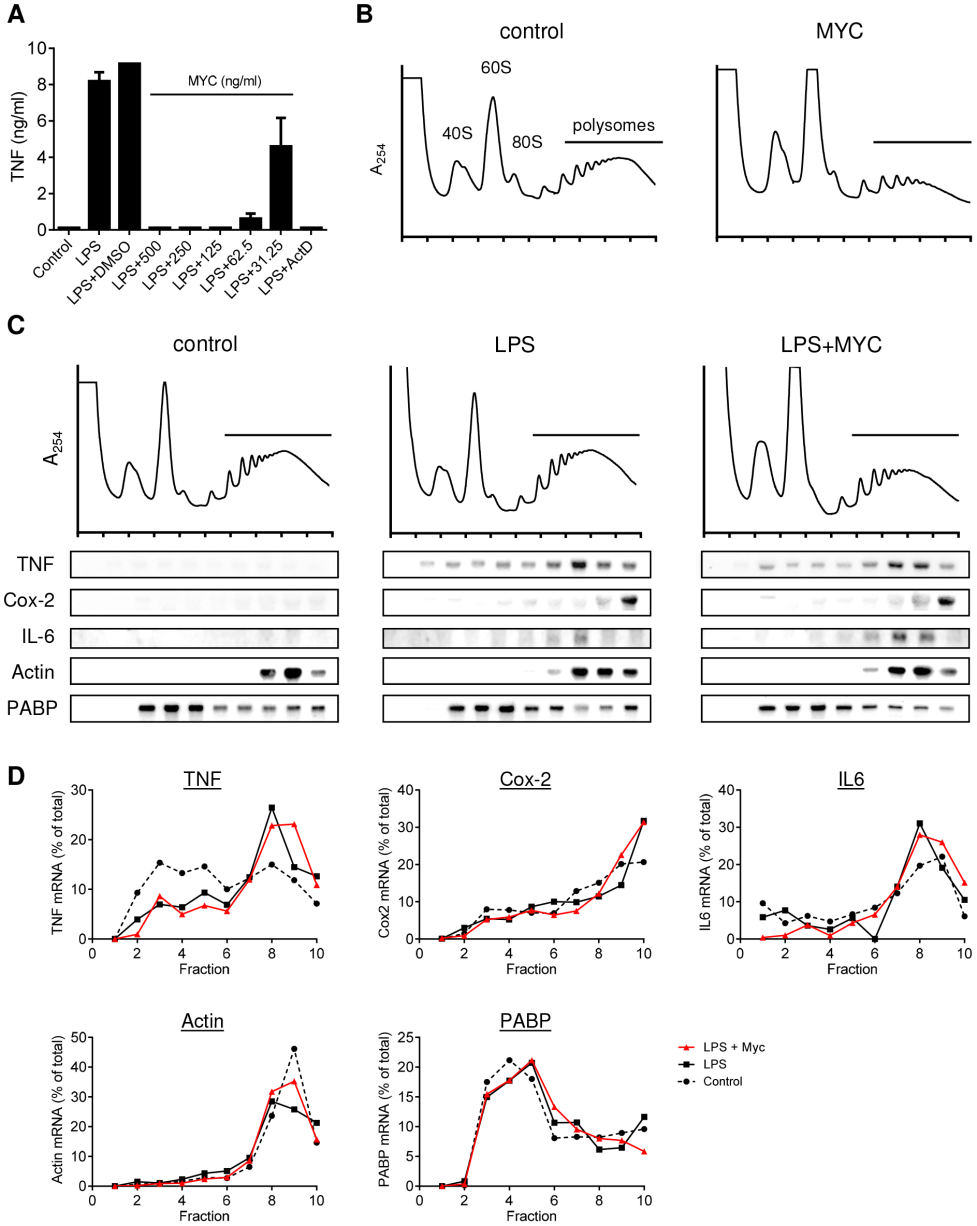
Mycolactone does not change the polysomal association of proinflammatory mRNAs. A. RAW264.7 cells were incubated for 1+/−various concentrations of mycolactone (MYC as indicated), 0.5 µg/ml Actinomycin D (Act D) or 0.0125% DMSO then stimulated or not with LPS for 4 hr. Supernatant TNF levels were measured by ELISA (mean±SEM of triplicate assays). B–D. RAW264.7 cells were incubated for 1 hr+/−125 ng/ml mycolactone (MYC) then stimulated or not with LPS. After 4 hrs cells were harvested and lysed in the presence of CHX. B and C. Polysomes were separated on a 10–50% sucrose gradient and the profiles measured by absorbance at 254 nm. Note the increase in the 60S peak and reduced height of the polysome peaks in MYC and LPS+MYC samples. RNA was purified from gradient fractions and transcripts were detected by Northern blotting using full coding region cDNA probes for the genes indicated. D. Signal intensity was quantified by ImageJ analysis of non-saturated phosphorscreen images. Values are presented as percentage of total signal for control (dashed line), LPS (solid black line) and LPS+MYC (red line) cells. All are representative of 3 independent experiments. PABP; poly A-tract binding protein.

In order to establish the dose of synthetic mycolactone A/B required to completely inhibit the production of TNF in RAW264.7 cells, we carried out a dose response (Fig. 1A). It was determined that the effective dose was 125 ng/ml, and this also prevented LPS-dependent Cox-2 production without affecting cell viability (Fig. S1A). This dose is marginally higher than required for inhibition of TNF production by natural mycolactone A/B in primary human macrophages (Fig. S1B), probably reflecting the known variation in sensitivity between different cell types, preparations of mycolactone (natural vs. synthetic) and/or target activities (immunosuppressive vs. cytotoxic). We then performed polysome profiling of macrophages to investigate whether mycolactone selectively inhibits the translation of inflammatory mediators. This technique allows the association of TNF, IL-6 and Cox-2 transcripts with actively translating polysomes in various experimental conditions to be assessed. RAW264.7 cells were used because, in preliminary experiments, the low mRNA yields and high RNase content of primary human monocytes and macrophages precluded the use of these cells (data not shown). The post-transcriptional mechanism of mycolactone-dependent inhibition of cytokine production observed in primary cells is conserved in this cell line (Fig. S1C, performed as a control experiment for all profiles obtained).

Mycolactone exposure was found to consistently cause a change in the shape of the polysome profiles, associated with an increase in the size of the 60S peak and change in the profile in the area associated with heavy polysomes in both unstimulated (Fig. 1B; MYC) and stimulated (Fig. 1C; LPS+MYC) cells. However, these changes occurred gradually over several hours, while the inhibition of TNF production is manifest as little as 20 min after LPS addition (data not shown). This suggests it may be a secondary, rather than primary, effect. Mycolactone alone did not influence the quantity or location of TNF mRNA (not shown) and LPS stimulation in itself did not induce any gross changes to the polysome profiles (Fig. 1C). In each profile, poly-A tract binding protein (PABP) and β-actin are used as control transcripts that confirm the location of unformed ribosomes and polysomes, respectively (Fig. 1C).

While unstimulated RAW264.7 cells expressed very little TNF mRNA, as expected, LPS stimulation led to increased abundance of TNF, IL-6 and Cox-2 mRNAs and their location moved so that a higher proportion of the mRNAs were in the polysomal fractions (Fig. 1C, compare ‘control’ and ‘LPS’ - and quantified in Fig. 1D), due to the known translational derepression that occurs following stimulation [21]. Again, as expected, neither the location of β-actin (known to be mycolactone insensitive [5]) or PABP were affected by mycolactone (Fig. 1B and D). However, in stark contrast to expectations, mycolactone had no effect on the polysomal association of any of the three inflammatory transcripts; all remained in heavy-polysomal fractions (Fig. 1B, compare ‘LPS’ and LPS+MYC’). When quantitated, the distribution of the mRNAs was very similar in the presence and absence of mycolactone (Fig. 1D).

**Figure 2:**
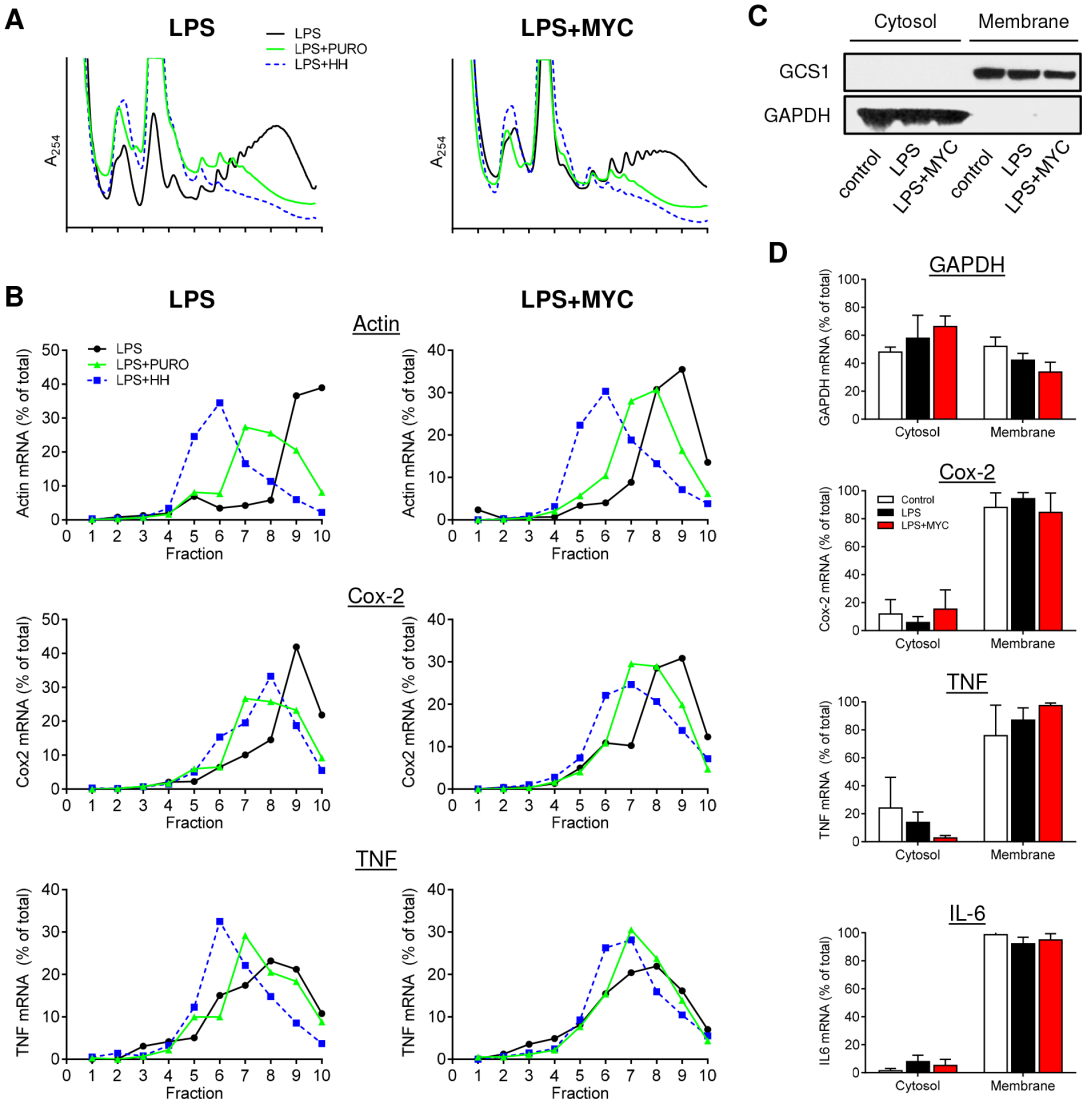
Proinflammatory mRNAs are actively translating in the presence of mycolactone. RAW264.7 cells were incubated for 1+/−125 ng/ml mycolactone (MYC), then stimulated with LPS. A and B. After 4 hr 100 µg/ml puromycin (PURO) or 5 µM homoharringtonine (HH) were added for 3 min, then CHX was added before lysis and separation of polysomes on a 10–20% sucrose gradient. LPS (solid black line), LPS+PURO (solid green line) and LPS+HH (dotted blue line). A. RNA profiles measured by absorbance at 254 nm. B. Quantitation of specific mRNAs purified from each fraction and analysed by Northern blotting. Signal intensity was quantified by ImageJ analysis of non-saturated phosphorscreen images. Values are presented as percentage of total signal. C and D. Cytosolic and digitonin-resistant membrane fractions were prepared from treated cells as described. C. Western blot of cell fractions (0.5×10^5^ cell equivalents/lane). GCS1; glucosidase I (∼92 kDa), GAPDH (∼40 kDa). D. total RNA was used as a template in qRT-PCR absolute quantitation assays and is presented as % total RNA for each gene (mean±SEM). All data representative of 3 independent experiments.

We confirmed this unexpected finding in a number of ways. First, the localisation of these transcripts was assessed at various times after LPS stimulation to investigate whether the findings were influenced by the kinetics of the LPS response or time of mycolactone exposure (>1 hr), but this was found not to be the case (data not shown). Second, we examined the effects of short term exposure to two translation-inhibiting drugs on polysome profiles (Fig. 2A and B). Puromycin (PURO) causes premature termination and ribosomal release from translating mRNAs, whereas homoharringtonine (HH) prevents translation initiation leading to ribosome run-off of translating mRNAs) [22], [23]. Neither drug influenced the production of TNF or its inhibition by mycolactone (Fig. S2), but both caused a change in the profiles obtained from LPS stimulated cells, with HH being the more efficient (Fig. 2A). As expected, there was a concomitant change in the location of β-actin mRNA to monosomes (HH) or lighter polysomes (PURO) (Fig. 2B, left panel LPS, compare the black with the blue or green lines respectively). Cox-2 and TNF mRNAs also both moved into lighter polysomal fractions, confirming that our experimental system was sensitive to inhibition of translation.

When the action of these drugs on mycolactone treated cells was assessed, it could be seen that, while mycolactone altered the profiles but not the location of β-actin, Cox-2 or TNF transcripts as before (Fig. 2A and 2B, black lines), the response to PURO and HH was the same in the absence and presence of mycolactone. For Cox-2, PURO caused a similar ∼2-fraction shift (Fig. 2B, green lines), whereas HH causes a similar ∼1-fraction shift (Fig. 2B, blue lines) in both untreated and mycolactone treated cells. It is interesting to note that the shift in the Cox-2 peaks following HH treatments were smaller than that seen for β-actin, suggesting that Cox-2 is being translated more slowly (compare the blue lines in Fig. 2B, LPS, Actin and Cox-2). Both drugs had a less marked effect on TNF but a movement of the peak of mRNA recovery to lower fractions could be seen that was not prevented by mycolactone. This shows that all of the tested mRNAs are undergoing active translation in both the presence and absence of mycolactone and are not stalled on the ribosomes.

Finally, in an independent approach, the cellular localisation of proinflammatory mRNAs in the presence of mycolactone was also examined. Since TNF and IL-6 are secreted proteins and Cox-2, is ER-resident and contains a signal peptide, their actively-translating, nascent polypeptide chains should be directly associated with the ER due to the interaction of the signal peptide with the signal recognition particle (SRP) and Sec61 complex [24]. Cells were selectively permeabilised with digitonin to separate the cytosolic and digitonin-resistant ER membrane fractions. Western blotting showed the presence GAPDH protein in the cytosol while the ER-resident protein glucosidase I (GCS1) was confined to the membrane fraction (Fig. 2C). As seen by others, GAPDH mRNA was fairly evenly distributed between cytosolic and membrane fractions [25], [26], but the mRNAs for TNF, Cox-2 and IL-6 were all predominantly in the membrane fraction, even in the presence of mycolactone, indicating sufficient synthesis had occurred to allow signal peptide recognition (Fig. 2D). This data also strongly argues against an inhibition of proinflammatory mRNA translation as the mechanism underlying the loss of protein production due to mycolactone.

### Inhibition of the 26S proteasome allows the cellular expression of pro-TNF and Cox-2 in the presence of mycolactone

**Figure 3:**
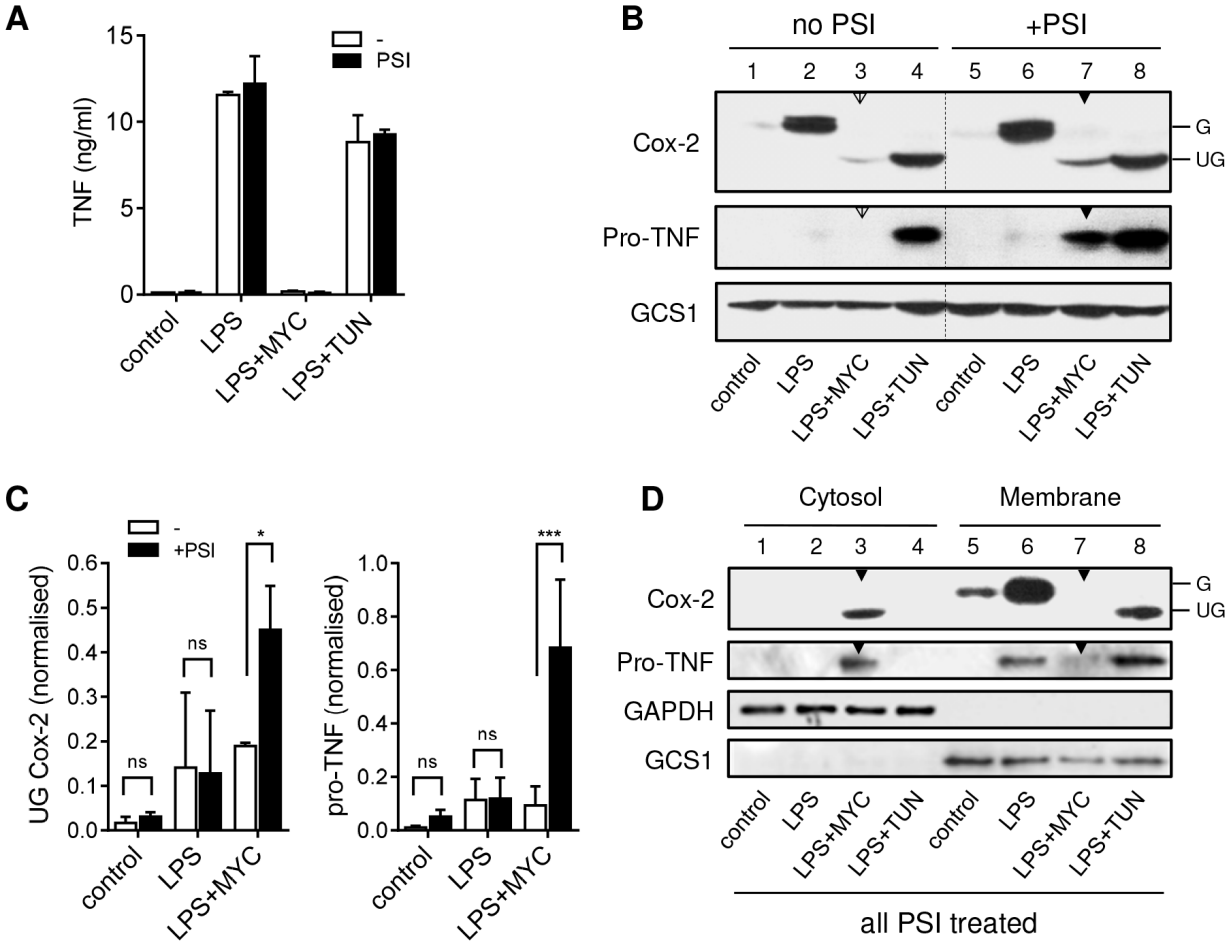
Mycolactone causes degradation of TNF and Cox-2 by the 26S proteasome in the cytosol. RAW264.7 cells were incubated +/−125 ng/ml mycolactone (MYC) or 5 µg/ml tunicamycin (TUN) for 1 hr and stimulated with LPS for 4 hrs. In certain samples 5 µM PSI was added 2 hrs after the LPS stimulation. This allowed time for the proteasome-dependent activation of NFκB required for transcriptional activation to occur before proteasome activity was inhibited (see text). A. Supernatant TNF levels from cells treated as above, measured by ELISA (mean±SEM). B. Western blot of cell lysates (0.5×10^6^ cell equivalents/lane), the dotted line separates samples with and without PSI treatment for ease of interpretation. C. Signal intensity was quantified by ImageJ analysis of non-saturated blots and normalised to LPS+TUN (not shown). Values are mean intensity for each lane±SEM of 3 independent experiments. For Cox-2 this is the unglycosylated mol wt band only (UG Cox-2). *, P<0.05; ***, P<0.001. D. Western blot of cytosolic and digitonin-resistant membrane fractions from cells treated with PSI as described above. All data representative of (or pooled from, C) 3 independent experiments. G; glycosylated Cox-2 (∼80 kDa), UG; unglycosylated Cox-2 (∼69 kDa), pro-TNF (∼26 kDa), GAPDH (∼40 KDa), GCS1; glucosidase I (∼92 KDa). Open arrowheads indicate cells treated with mycolactone but not PSI. Closed arrowheads indicate cells treated with mycolactone and PSI.

As proinflammatory protein synthesis is maintained in the absence of detectable protein levels, mechanisms by which these proteins might be targeted for degradation by the cell were investigated. Degradation by the 26S proteasome seemed a likely candidate. However, examining this experimentally is complex for inflammatory mediators since their transcriptional activation requires proteasome-dependent degradation of IκBα [27]. Cells were therefore stimulated with LPS for 2 hrs prior to addition of the proteasome inhibitor (PSI) for an additional 2 hrs. This did not decrease LPS-dependent production of TNF in cell supernatants (Fig. 3A) indicating that this experimental design was satisfactory.

Treatment of RAW264.7 cells with mycolactone resulted not only in a profound decrease in LPS-dependent Cox-2 production, but the barely detectable immunoreactive protein also had a lower mol wt (Fig. 3B, compare lanes 2 and 3), equivalent to that seen in cells exposed to the N-glycosylation inhibitor tunicamycin (TUN; Fig. 3B lane 4). Remarkably, Cox-2 production was found to increase when 26S proteasome activity was blocked in mycolactone-treated cells (Fig. 3B, compare lanes 3 [open arrow] and 7 [closed arrow]). When this effect was quantified by densitometry (Fig. 3C), then considered as fold-change within each experiment, the extent of restoration of protein production was 2.31±0.35-fold (mean±SEM, P = 0.02, n = 3). Notably, this protein was also unglycosylated (Fig. 3B, Cox-2 lane 7 [closed arrow]).

An even more striking observation was made for TNF, detected as nascent pro-TNF (26 kDa) within cell lysates. In LPS-stimulated cells pro-TNF is rapidly exported and so is barely detectable in cell extracts (Fig. 3B lane 2). However, while the low-level ER stress induced by TUN does not reduce TNF in cell supernatants (Fig. 3A) it does slows transit through the ER and Golgi allowing detection of pro-TNF in lysates [28] (Fig. 3B, lane 4). In the absence of PSI, no pro-TNF could be observed following mycolactone treatment, but levels of pro-TNF increased dramatically upon inhibition of 26S proteasome activity (Fig. 3B, compare lanes 3 [open arrow] and 7 [closed arrow]). When this effect was quantified by densitometry and analysed as for Cox-2 (Fig. 3C) this equated to a mean fold-increase of 12.24±6.6-fold (mean±SEM, P = 0.0002, n = 3). This is the first time that the inhibition of cellular TNF and Cox-2 production has been overcome in the continuous presence of inhibitory concentrations of mycolactone, allowing detection of the previously undetectable protein. This transformative finding supports a mechanism for mycolactone action in which such proteins (including, but not restricted to, TNF and Cox-2) are being destroyed by proteosomal degradation in the cytosol.

### Mycolactone inhibits co-translational translocation into the ER

**Figure 4:**
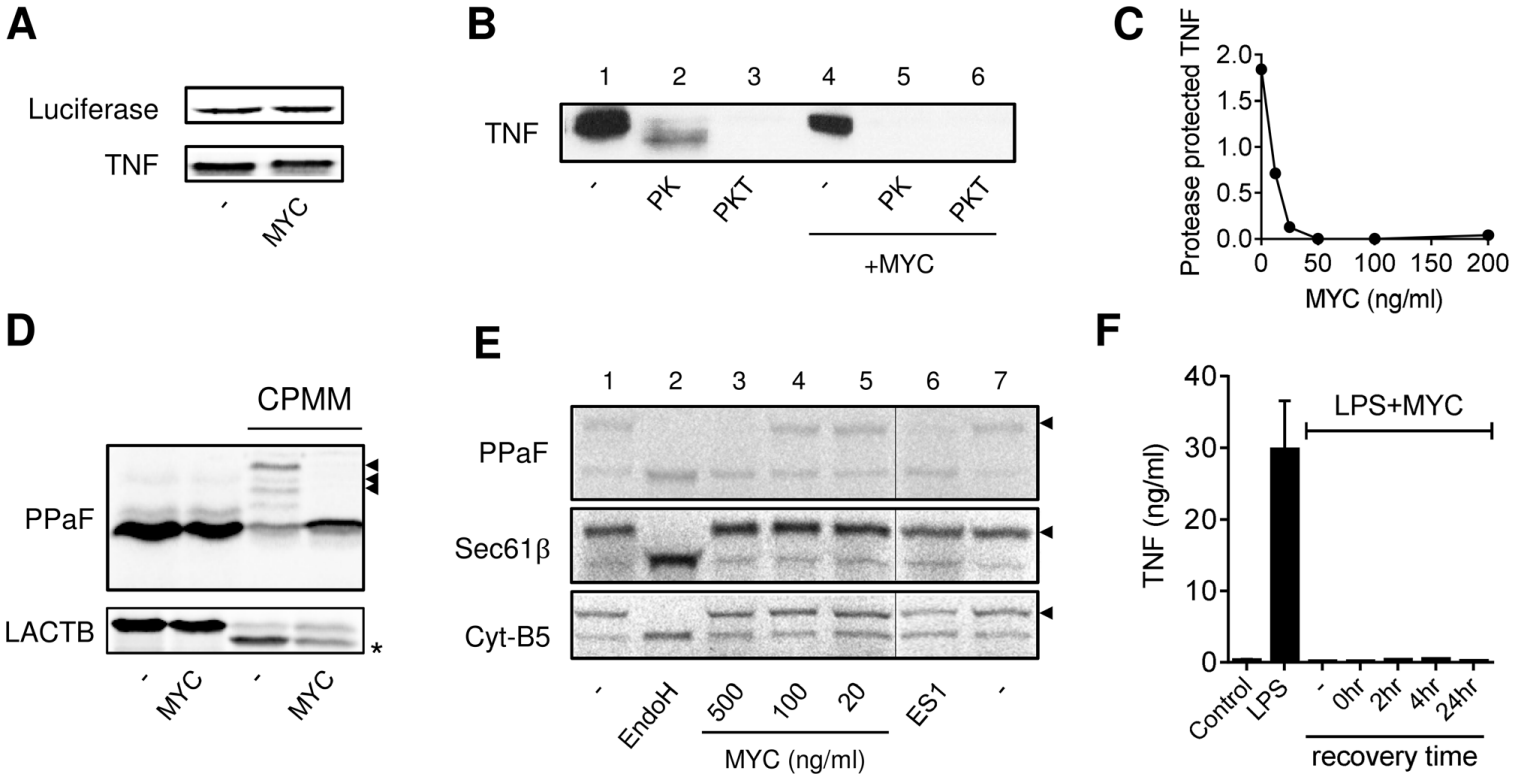
Mycolactone inhibits co-translational translocation of proteins into the ER via a mechanism that does not disrupt the structural integrity of the ER. A–E. *In vitro* translation (IVT) reactions of different capped transcripts were performed as described +/− mycolactone (MYC; 200 ng/ml unless indicated otherwise), Eeyarestatin 1 (ES1; 250 µM) or DMSO (-; 0.02%). Data representative of 3 independent experiments. A. IVT of luciferase and TNF mRNAs detected by ^35^S incorporation and Western blot (∼26 kDa) respectively. B. IVT of TNF mRNA was performed in the presence of semi-permeabilised RAW264.7 cells then incubated with no addition (-), or Proteinase K (PK) +/−0.1% Triton-x-100 (PKT) for 1 hr at 4°C before stopping the reaction. C. Dose dependence of loss of the PK protected band. Signal intensity was quantified by ImageJ analysis of non-saturated blots and the protected band (PK) was normalised to total pro-TNF (-). D. IVT of prepro-α Factor (PPAF) and β-lactamase (LACTB) mRNAs in the absence or presence of canine pancreatic microsomal membranes (CPMM). Black arrowhead, glycosylated forms of α Factor; *, signal peptide-cleaved LACTB. E. IVT of PPAF, Sec61β and cytochrome B5 (Cyt-B5) mRNAs in the presence CPMM. After labelling with ^35^S as described, membranes were isolated by centrifugation through a sucrose cushion [31]. Endoglycosidase H (EndoH) is used to confirm glycosylation of proteins (black arrowhead), the thin line represents where an empty lane was removed from the image for ease of interpretation. F. RAW264.7 cells were incubated +/−125 ng/ml mycolactone (MYC), washed and incubated without mycolactone for various periods (recovery time) before stimulating with LPS for 4 hrs. Supernatant TNF levels were measured by ELISA (mean±SEM of triplicate assays).

The lack of glycosylation of Cox-2 and the failure of PSI treatment to cause TNF secretion (Fig. 3A), suggested the restored proteins could not gain access to the ER. Indeed, after digitonin permeabilisation of PSI-treated cells, unglycosylated Cox-2 and pro-TNF were predominantly found in the cytosolic fraction of mycolactone treated cells (Fig. 3D, lane 3 [closed arrow]), in contrast to the LPS-induced proteins that were glycosylated and membrane associated (Figure 3D lane 6). Unglycosylated Cox-2 was found in the membrane fraction after TUN treatment showing that lack of N-glycosylation alone was insufficient to explain its localisation in mycolactone-treated cells (Fig. 3D lane 8). Inhibition of the Sec61 translocon by small molecule inhibitors, rather than causing accumulation of proteins intended for export in the cytoplasm, tends to trigger rapid degradation of the mislocalised proteins [29], [30], similarly to mycolactone. We therefore assessed whether the degradative loss we observe could be due to a blockade of translocation, using assays in which different mRNAs (transcribed and capped *in vitro*) undergo *in vitro* translation (IVT) in the absence or presence of ER containing cellular membrane preparations (Fig. 4). When such membranes are absent, no modifications of the proteins are possible. However, when membranes are present, the nascent proteins produced can undergo co-translational translocation into the ER via the Sec61 translocon and can consequently be either glycosylated or processed to remove the signal peptide sequence. In agreement with our other findings, mycolactone had a minimal direct effect on the synthesis of TNF in both the absence (Fig. 4A; TNF and luciferase mRNAs) and presence (Fig. 4B, TNF compare lanes 1 and 4) of membranes provided by semi-permeabilised RAW264.7 cell extracts.

In order to test whether TNF could co-translationally translocate into the ER in the presence of mycolactone we used Proteinase K, which can only digest proteins that it can access (i.e. those outside of the added membranes). A proportion of newly synthesised TNF could be protected from Proteinase K digestion, resulting in a resistant band of slightly lower mol wt (due to loss of pro-TNF's cytoplasmic tail, Fig. 4B, lane 2). This protected fragment was lost on inclusion of detergent (Fig. 4B, lane 3), as expected, since the Proteinase K could now access proteins in the internal membrane compartment. Mycolactone efficiently prevented TNF from translocating into the protected membrane compartment since its addition led to a complete loss of the Proteinase K resistant band (Fig. 4B, compare lanes 2 and 5). This inhibition of translocation was dose-dependent (IC_50_≈15 nM, Fig. 4C).

A complete blockade of TNF translocation into the ER is necessary and sufficient to explain the loss of TNF production and rapid degradation we have observed. Co-translational translocation is a mechanism utilised by many proteins, and there are some well-established model precursor proteins that can be used to investigate whether mycolactone's inhibition is more generally applicable. The N-glycosylation of yeast prepro-α Factor (PPaF) is dependent on the addition of canine pancreatic microsomal membranes (CPMM, Fig. 4D) and can be reversed by the deglycosylating enzyme Endoglycosidase H (EndoH, Fig. 4E). Mycolactone reproducibly blocked PPaF from being glycosylated (Figs 4D and 4E), and this mimicked the activity of another known translocation inhibitor, Eeyarestatin 1 (ES1, Fig. 4E). Whilst 250 µM ES1 is typically employed to inhibit ER translocation *in vitro* [31], much lower levels of mycolactone (0.25–0.7 µM) achieved a comparable effect (Figs. 4D and 4E; PPaF). The precise concentration of mycolactone required for a complete inhibition of *in vitro* translocation may be influenced by the amount of ER derived membranes present in the assay (cf. Figs 4D and 4E). Likewise, the cleavage of the β-lactamase signal peptide can also be detected after the addition of CPMM, providing an alternative measure of ER translocation. In this case we observed a substantial reduction in signal sequence cleavage although the effect was not complete (Fig. 4D, LACTB).

Mycolactone is a lipid-like molecule that is reportedly present in the cytoplasm of treated cells [8], [32], but an inhibition of translocation suggests that it may be interacting with the ER membrane in some way. Cells that are allowed to recover for 24 hr after 1 hr of mycolactone exposure are still unable to produce TNF, suggesting that this activity is irreversible (Fig. 4F). To determine whether mycolactone mediates its functions simply by disrupting organelle membrane structures we studied whether mycolactone could prevent the insertion of tail-anchored membrane proteins into the ER. Such proteins, including the β subunit of the Sec61 complex (Sec61β) and cytochrome B5 (Cyt-B5), are inserted into the ER membrane in a post-translational, Sec61-independent, manner [33], and are subsequently glycosylated on artificial C-terminal reporters (Fig. 4E, compare lanes 1 and 2). While mycolactone prevented the *in vitro* glycosylation of PPaF, neither mycolactone nor ES1 affected the N-glycosylation of Sec61β or Cyt-B5 (Fig. 4E, lanes 3 and 6), ruling out an effect on either membrane integrity or the N-glycosylation machinery. In addition, transmission electron microscopy showed that mycolactone did not disrupt the ultrastructure of LPS-stimulated RAW264.7 (data not shown). On this basis we conclude that the loss of co-translational translocation into the ER resulting from treatment with mycolactone reflects a specific blockade of the membrane translocation machinery.

### Mycolactone-dependent inhibition of translocation is not accompanied by ER-associated degradation (ERAD), ER stress or WASP activation

**Figure 5:**
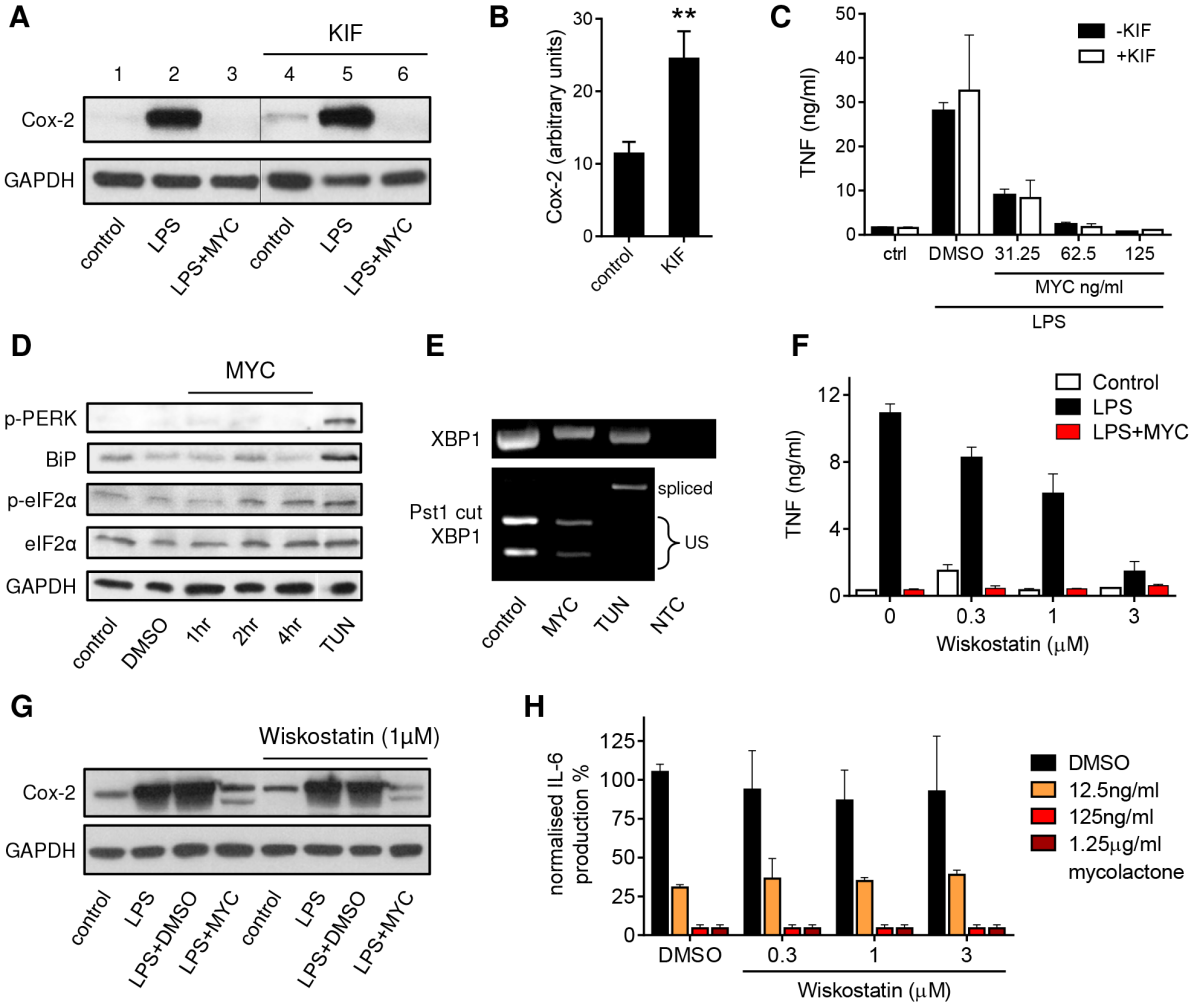
Mycolactone does not mediate these effects via ERAD, ER stress or WASP-activation-dependent mechanisms. A–C. RAW264.7 cells were incubated +/−125 ng/ml mycolactone (MYC), +/−50 µM Kifunensine (KIF) or 0.0125% DMSO for 1 hr and stimulated or not with LPS for 4 hrs as indicated. A. Western blot of cell lysates (0.5×10^6^ cell equivalents/lane); the thin line represents where an empty lane was removed from the image for ease of interpretation. B. Quantitation of constitutive expression of Cox-2 in lanes 1 and 4 (control and KIF respectively) Pixel intensity was determined using ImageJ software and normalised according to GAPDH. Results represent the mean±SEM (n = 3). C. Supernatant TNF levels measured by ELISA (mean±SEM). D. RAW264.7 cells were incubated +/−125 ng/ml mycolactone (MYC) for various periods or 5 µg/ml tunicamycin (TUN) or 0.0125% DMSO for 4 hrs. Western blot of cell lysates (0.5×10^6^ cell equivalents/lane). E. Total RNA of treated/unstimulated cells was used as a template for RT-PCR of XBP-1 (upper panel) which was then digested with *Pst1* and separated on a 1% agarose gel (lower panel). NTC; no template control, US; unspliced. F–G. RAW264.7 cells were incubated +/−125 ng/ml mycolactone (MYC), −/+ wiskostatin at a range of concentrations or 0.0125% DMSO for 1 hr and stimulated or not with LPS for 4 hrs. F. Supernatant TNF levels measured by ELISA (mean±SEM). G. Western blot of cell lysates (0.5×10^6^ cell equivalents/lane). H. HeLa cells were incubated with a range of doses of mycolactone (MYC) and wiskostatin or 0.0125% DMSO for 1 hr and stimulated or not with 10 ng/ml IL-1β. IL-6 in supernatants was measured by ELISA (mean±SEM of production, normalised to IL-6 production in otherwise untreated, IL1β stimulated cells). Cox-2 (∼90 kDa), GAPDH (∼40 kDa), p-PERK (140 kDa), BiP (∼78 kDa), (p-)eIF2α (∼38 kDa).

While inhibition of translocation across the ER is sufficient to explain the loss of inflammatory mediators by mycolactone, we also investigated whether other cellular mechanisms might also contribute. First, we examined whether it might activate ER-associated degradation (ERAD) since this pathway can recognise unfolded proteins, deglycosylate and then degrade them in a ubiquitin-dependent manner. Specifically we asked whether Kifunensine (KIF; a class I α-mannosidase inhibitor and a well-established suppressor of ERAD [34], [35]) could overcome mycolactone-dependent inhibition of protein production reasoning that, if this was the case, then KIF treatment should restore protein production. The biological activity of KIF in this system was confirmed by monitoring the expression of constitutive Cox-2 expression (Fig. 5A, compare lanes 1 and 4) in the absence of LPS or mycolactone, since this is known to be turned-over by ERAD [36]. KIF significantly increased Cox-2 expression under these conditions (Fig. 5B), so we can be sure that it does inhibit ERAD at this dose in RAW264.7 cells. However, KIF was unable to restore either production of Cox-2 (Fig. 5A, compare lanes 3 and 6) or TNF secretion (Fig. 5C) in mycolactone-treated cells. This argues against mycolactone being an activator of ERAD-dependent ER export.

In addition, as shown previously in primary human monocytes [5] and T cells [17], eIF2α was not significantly phosphorylated in mycolactone-treated RAW264.7 cells (Fig. 5D). Moreover, it did not cause the phosphorylation of PERK, induce expression of BiP (Fig. 5D) or cause the IRE-dependent splicing of XBP-1 (Fig. 5E), in contrast to the known inducer of ER stress, tunicamycin (TUN). Therefore ER stress, as defined by conventional markers, cannot explain mycolactone action.

Recently, inappropriate activation of WASP family proteins by mycolactone was shown to lead to changes in cell adhesion and migration, some of which are reversed by wiskostatin [8], an inhibitor of N-WASP GTPase activity. Since actin dynamics might conceivably contribute to the inhibition of TNF and Cox-2 production by mycolactone, as well as cell adhesion, the effect of wiskostatin in our system was investigated. In contrast to previous reports in human macrophages [37], wiskostatin itself had an inhibitory effect on TNFα secretion in RAW264.7 cells (Fig. 5F). When co-incubated with mycolactone it could not restore protein production. This included the loss of LPS-induced TNF (Fig. 5F) and Cox-2 (Fig. 5G) in RAW264.7 cells and the IL1β-induced production of IL-6 (Fig. 5H) and IL-8 (data not shown) in HeLa cells at our inhibitory dose of 125 ng/ml. Since the reported restorative effect of wiskostatin on HeLa cell adhesion was determined using a lower dose of natural mycolactone A/B [8] we examined wiskostatin's effect at a range of doses of both mycolactone and wiskostatin, but could find no evidence to support an influence of WASP inhibition over cytokine production (Fig. 5H).

### Mycolactone prevents the production of the vast majority of N-glycosylated and secreted proteins that transit through the ER

**Figure 6:**
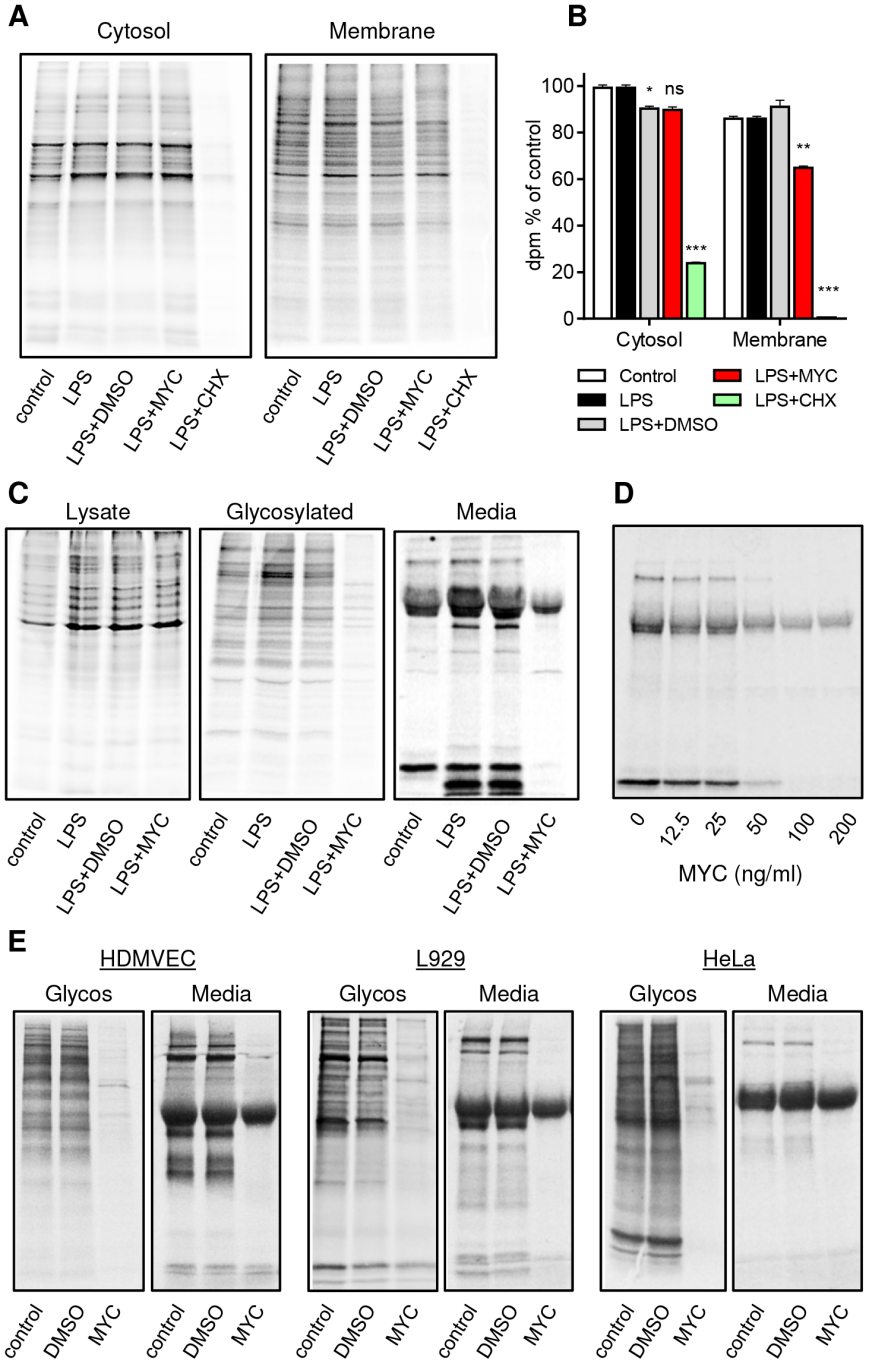
Mycolactone specifically targets membrane and secreted proteins. Cells in Met/Cys free medium were incubated +/−125 ng/ml mycolactone (MYC), 10 µg/ml cycloheximide (CHX) or 0.0125% DMSO for 1 hr and stimulated with LPS for 1 hr before replacement with fresh medium containing Tran35slabel for 2 hrs. Data representative of 3 independent experiments (A–D). A. Cytosolic and digitonin-resistant membrane fractions from treated RAW264.7 cells (10^5^ cell equivalents/lane). B. Quantification of ^35^S incorporation in (A) by scintillation counting (mean±SEM, n = 3). *, P<0.05; **, P<0.01; ***, P<0.001. C. Total cell lysates, Concanavalin A (ConA) agarose precipitated proteins (Glycosylated) and supernatants (Media) from RAW264.7 cells. D. Dose dependence of the suppression of supernatant protein production in RAW264.7 cells. E. ConA precipitated (Glycos) and supernatant proteins (Media) from ^35^S labelled Human microvascular dermal epithelial cells (HDMVEC), L929 fibroblasts and Hela cells. In each case total cell labelling was comparable between samples (not shown).

Translocation of proteins into the ER is a widespread cellular phenomenon but is nevertheless restricted to a defined subset of proteins, most of which carry a canonical signal peptide. Our findings suggest that in mycolactone-exposed cells, the production of most proteins within this subset may be blocked. We tested this by examining protein synthesis in different cellular compartments (Fig. 6A and B). The translation elongation inhibitor cycloheximide (CHX) led to an almost complete block in protein synthesis in both cytosolic and membrane fractions of RAW264.7 cells, as expected. Yet, while mycolactone caused little change in cytosolic protein synthesis, it did cause a selective ∼30% decrease in membrane-associated proteins (Fig. 6A and B, P<0.01). Since the membrane fraction would include proteins from various intracellular organelles (nucleus, mitochondria etc) as well as exported proteins, Concanavalin A (ConA) agarose was used to isolate glycosylated proteins in order to better represent the subset of proteins that must translocate into the ER. As predicted, a large decrease in the recovery of such constitutive and induced proteins from mycolactone-treated cells was found (Fig. 6C). Notably, the degree of loss varied and a minority of proteins showed little or no change. When supernatants were examined, the abundance of almost all constitutive and LPS-induced proteins was reduced, with only a single ∼32 kDa protein unaffected (Fig. 6C). This was dose-dependent (Fig. 6D) and production of most mycolactone-sensitive proteins showed similar IC_50_ values (57.8±7.9 nM, mean±SEM, n = 3). Indeed, when we investigated the production of 18 cytokines and chemokines produced by RAW264.7 cells (Fig. S3A), we found that 17 were almost completely blocked by mycolactone after LPS stimulation (Fig. S3B and C). Those not reported as targets of mycolactone previously include TIMP-I, soluble ICAM-1 and IL-RA. A single chemokine, MIP-1α seemed to be completely insensitive to mycolactone inhibition in this antibody array, however validation by ELISA showed that these antibodies may be saturated by the high levels of constitutive protein, since this more quantitative analysis showed a profound inhibition by mycolactone (Fig. S6D). A similar phenomenon is most likely also responsible for the apparent reduced sensitivity of constitutively expressed JE (CCL2, commonly known as MCP-1 in humans) and MIP-1β in the absence of LPS (Fig. S3B and C), especially since others have previously shown that both are mycolactone targets in other systems [16], [18].

Since many different types of cells are exposed to mycolactone in BU lesions and translocation by the Sec61 complex is highly conserved [38], we examined whether the production of secreted and glycosylated proteins would be similarly prevented in non-immune cells. Interestingly this was found to be the case in human dermal microvascular endothelial cells (HDMVEC), murine L929 fibroblasts and HeLa cells (Fig. 6E). In all cases, there was a profound inhibition of protein production affecting the majority of proteins in these compartments. Therefore, a block in translocation of proteins into the ER represents a widely applicable mechanism underlying mycolactone's pathogenic effects.

## Discussion

In this manuscript we have identified an important new activity for the *M. ulcerans* virulence factor, mycolactone. By investigating the inhibition of cytokine production as a model system to examine its basic cell biology, we have shown that mycolactone effectively blockades the translocation of nascent proteins across the ER membrane in a mechanism that seems to involve the Sec61 translocon. This finding is remarkable because previous data suggested that mycolactone was probably inhibiting the translation of inflammatory mediators such as TNF, IL-6 and Cox-2 [2], [5]. However, our detailed investigation refutes this. We showed conclusively that the transcripts were actively translating *in vitro* and in a RAW264.7 cell model in both the absence and presence of mycolactone. The polysomal location of each of these transcripts in treated cells is remarkably similar to that seen in untreated cells and their relocation in response to inhibitors of translation is also unaffected. Likewise the demonstration of an association between these mRNAs and the membrane fraction in mycolactone-treated cells provides independent evidence of sufficient translation having occurred to allow signal peptide-directed targeting to the ER.

We provide multiple lines of evidence in support of mycolactone's ability to prevent translocation across the ER and induce subsequent degradation in the cytosol. This is supported first by the finding that translocation of TNF into a protease resistant membrane compartment could no longer occur in the presence of mycolactone *in vitro*. Furthermore, production of both Cox-2 and cellular pro-TNF was restored when the protease activity of the proteasome was blocked by PSI after transcriptional activation and translational derepression had occurred. The restored Cox-2 was unglycosylated; however this is most likely a consequence of never having entered the ER rather than a cause of degradation. The effects of TUN and mycolactone on TNF secretion and Cox-2 production are quite distinct and the restored Cox-2 and TNF were both present in the cytosol rather than the membrane. Therefore, while blocking the proteasome prevented the degradation of the proteins, it could not overcome mycolactone's translocation blockade and the restored proteins would not be able to carry out their normal cellular function. Proteasome inhibitors such as Bortezomib have attracted some attention as cancer therapeutics, but for the reasons laid out above such drugs should not be considered for BU treatment.

There are a number of mechanisms by which proteins can enter the ER and then go on to be secreted, retained in the ER or inserted into the membrane. These include co- and post-translational Sec61-dependent translocation as well as Get/TRC40-dependent insertion of tail anchored proteins, reviewed in [39]. Among cytokines, TNF is slightly unusual in that it is made as pro-TNF, co-translationally inserted into the ER membrane as a Type II membrane protein then cleaved at the cell surface (by TNF-converting enzyme; TACE). Most others, including IL-6, undergo conventional trafficking via post-translational translocation into the ER, followed by signal sequence cleavage. IL-1β and IL-18 provide notable exceptions to this rule, and are secreted by an unconventional mechanism following cleavage of the cytosolic pro-isoforms in a caspase- and inflammasome-dependent manner [40]. The precise cellular mechanism that results in consequent export of mature cytokines is still somewhat controversial. Neither IL-1β nor IL-18 can be made by RAW264.7 cells (see Fig. S3C) as they lack the required ASC (Apoptotic speck protein containing a caspase recruitment domain [41]), and so they were not studied further here. However, it is interesting to note that in our previous work with primary human monocytes, IL-1β production was only partially blocked by mycolactone (between 30–70% depending on the TLR ligand used to activate the cells [5]). The ability of mycolactone to inhibit unconventional secretion is now the subject of further investigation.

To our knowledge, mycolactone is the first and only virulence factor shown to inhibit translocation into the ER. A very few, non-pathogenic, compounds have been described that block Sec61-mediated transport: the substrate selective inhibitors cotransin (derived from the natural product HUN-7293) and its close relative CAM741, as well as ES1 and Apratoxin A. In each case a block in the cotranslational translocation of exported peptides leads to their rapid degradation in the cytosol [29]–[31], [42]. ES1 is arguably the best characterised of the translocation inhibitors but it must be used at a much higher dose (8 µM for treatment of cells, 250 µM in IVT) than is required for mycolactone-dependent inhibition. In a direct comparison, both were found to be similarly inactive against the insertion of tail-anchored proteins (Fig. 4E). In addition to its ability to inhibit entry into the ER, ES1 also inhibits ERAD driven exit and triggers an unfolded protein response ([43], [44] and data not shown). We investigated both these other pathways here. The inability of KIF to enhance expression of TNF or Cox-2 in the presence of mycolactone makes increased export of misfolded proteins via the ERAD pathway an unlikely mechanism contributing to the loss of inflammatory mediators (Fig. 5A and B). Moreover, we found no evidence of induction of the unfolded protein response in mycolactone-treated cells. Therefore mycolactone's cellular effects are similar to, but discrete from, ES1.

In contrast to mycolactone and ES1's ability to prevent the production of nearly all glycosylated and secreted proteins (Fig. 6C–E and [31]), cotransin is both non-toxic and highly selective in its action, affecting only a small subset of substrates [30]. Apratoxin A causes a wide inhibition of protein secretion but, also differs from mycolactone because its effects are completely reversible [42]. To date in the literature, there have been contradictory reports on the reversibility of mycolactone action. While L929 fibroblasts are reported to regrow after the removal of mycolactone [1], dendritic cells cannot regain the ability to respond to maturation stimuli after a prolonged exposure (24 hrs) [16]. Here we show that, for cytokine production, the effects remain irreversible after 24 hours, even after a brief exposure (Fig. 4F).

The recently reported ability of mycolactone to enhance actin polymerisation and inappropriately activate WASP [8] does not seem to be a major contributor to the inhibition of production of inflammatory mediators. Mutations in human WASP are associated with a range of immune dysfunction disorders and WASP has also been implicated in Golgi to ER transport [45], [46]. However, doses of the WASP inhibitor wiskostatin (equivalent or higher than those shown to partially restore adhesion in HeLa cells [8]) could not restore secreted protein production at a range of doses of mycolactone (Fig. 5F–H). Therefore, the decisive step in mycolactone-dependent inhibition of protein production seems to depend on the translocation blockade rather than WASP activation. Determining the precise mechanism by which mycolactone blocks translocation into the ER, and the molecular consequences of this action are the subject of ongoing investigation. It will be interesting to determine the molecular explanation of how some signal-peptide containing proteins including β-lactamase (Fig. 4D) and CCR7 [17] escape this translocation block. Indeed selectivity in mycolactone action has been fairly widely reported and the variable sensitivity to mycolactone inhibition observed in our *in vitro* translocation assays is consistent with this [5], [6], [16]–[18].

The broad spectrum inhibition of protein translocation induced by mycolactone in various cell types has important implications across diverse pathologies of Buruli ulcer. The suppression of innate and adaptive immune responses mediated by secreted cytokines and chemokines is one important aspect of this. In this work, we have identified nine novel immune proteins that are sensitive to mycolactone (BCA, MIP-2, G-CSF, GM-CSF, IL-27, C5/C5a, sICAM-1, IL-1RA and TIMP-1). In addition, the general block in production of glycosylated proteins would also affect many other proteins destined for the cell surface that mediate essential cellular functions. This would include those involved in aspects of adaptive immunity driven by maturation markers and costimulatory molecules on dendritic cells [16] where an inability to produce these proteins would lead to a failure to interact with lymphocytes, causing the T cell anergy seen in BU [2], [47], [48]. Likewise, the loss in T cell homing has been attributed to loss of receptor expression and this powerful mechanism of suppression would substantially add to the effect of let-7b [6]. These authors did investigate the role of proteasomal inhibition with MG132, but the experimental technique used (flow cytometry of fixed, permeabilised cells) would probably be insensitive to the restoration of protein that would now be located in the cytosol (see Fig. 3D) and therefore unlikely to be in its native conformation. In addition, the translocation block may contribute, along with WASP activation [8], to the rounding up and loss of adhesion seen in fibroblasts due to gradual loss of cell adhesion molecules as they turn over. Since anoikis is described as driving cell death, this could be an important contributory factor in the necrosis seen in ulcers. Moreover, a steady depletion of endogenous proteins in this way would lead to a gradual winding down of cellular function, rather than rapid cell death, which ties in well with the slow progression of the disease in humans, as well as *in vitro* and *in vivo* data [4]. For instance, the delay between injection of even 100 µg of mycolactone into guinea pig and ulcer formation is 5 days [1], although some early signs of apoptosis are evident from 2 days [7].

BU is the third most common human mycobacterial infection in the world after tuberculosis and leprosy. Although not fatal, patients can suffer lifelong disfigurement and disability unless the infection is recognised and treated at an early stage. Treatment with antibiotics is often accompanied by increased swelling and ulceration, or even the appearance of new lesions [49]. Such paradoxical responses are thought to arise from a gradual increase in proinflammatory activity as mycolactone levels decline. Several studies in both human patients and animal models have shown that a progressive and structured immune response follows initiation of therapy with the standard rifampicin/streptomycin combination [20], [50], [51]. As bactericidal activity does not always correlate with healing rate, it has been suggested that the drugs, rifampicin in particular, may inhibit the ability of *M. ulcerans* to synthesise mycolactone and that this is the more important factor in recovery [51], [52]. It seems unlikely that this phenomenon is due to a direct effect of the antibiotics on the immune system. Although rifampicin is reported to be immunomodulatory, responses vary and while some proinflammatory responses are enhanced by exposure to the drug, others are blocked [53], [54]. The molecular mechanisms of these effects seem independent from Sec61-dependent translocation and are mostly upstream of this process. In Jurkat T cells for example, rifampicin inhibits TNF production by blocking NFκB activation [54]. However, the possibility that antibiotics may promote restoration of immune function by altering the interaction between mycolactone and its cellular target cannot yet be ruled out and is worth investigating. A greater understanding of these interactions would give insights valuable to improving diagnosis and treatment of this debilitating disease. Furthermore, unravelling the mechanisms of mycolactone activity should also yield novel insights into the vital, basic cellular process of ER translocation.

## Materials and Methods

### Reagents

A full list of reagents, primers and antibodies can be found in Information S1. We used synthetic mycolactone A/B (kind gift of Prof. Yoshito Kishi, Harvard University) throughout these investigations [55]. All reagents used in tissue culture were routinely tested for endotoxin contamination by LAL assay (Lonza) and were <0.1 U/ml LPS.

### Cell culture and stimulation

The RAW264.7 murine macrophage cell line (ATCC) was routinely cultured at 37°C and 5% CO_2_ in high glucose DMEM medium (PAA) supplemented with 10% FBS (Life Technologies). Culture conditions for additional cell types can be found in Information S1. Cells were pre-incubated with mycolactone or other inhibitors for 1 hr before stimulation with 100 ng/ml TLR-grade LPS (Enzo Life Sciences) then incubated for 1–4 hr before harvesting of cells and culture supernatants. Mycolactone was routinely used at a final concentration of 125 ng/ml; DMSO diluted to the same extent (0.0125%) was the control. Other inhibitors used were PSI (5 µM, Calbiochem), CHX (10 µg/ml), ActD (2 µg/ml), KIF (50 µM), TUN (5 µg/ml), and wiskostatin (1 µM). For proteasome dependent degradation experiments, cells were pre-incubated with mycolactone or TUN and stimulated with LPS for 2 hr, prior to the addition of PSI for a further 2 hr. To investigate the reversibility of mycolactone action, cells were exposed to mycolactone for 1 hr, then mycolactone was removed, the cells were washed with PBS then incubated in complete DMEM for the indicated time, after which cells were stimulated with LPS for 4 hrs before harvesting the supernatants. HeLa cells were stimulated with 10 ng/ml recombinant IL-1β (Peprotech).

### Polysome profiling

Polysome profiling by sucrose density gradient ultracentrifugation was carried out according to a previously published method [56], [57]. A full protocol can be found in Information S1. Briefly, treated RAW264.7 cells were harvested 10 min after ribosomes were stalled with CHX (10 µg/ml). In some cases, 100 µg/ml PURO or 5 µM HH were added 3 min prior to addition of CHX. Cell lysates were separated over a 10–50% sucrose gradient. RNA was extracted from 1 ml fractions and analysed by Northern blotting using ^32^P-labelled (full coding region) cDNA probes.

### Digitonin permeabilisation

Membrane bound and cytosolic cellular fractions were separated by digitonin permeabilisation using a previously published method [26] with minor alterations (full protocol in Information S1). Briefly, proteins from treated RAW264.7 cells were extracted by sequential use of permeabilisation (0.03% digitonin) and solubilisation (1% NP-40, 0.5% sodium deoxycholate) buffers.

### *In vitro* translation

TNFα cDNA was prepared from LPS-stimulated primary human monocytes by RT-PCR and capped mRNA synthesised using Message Machine (Applied Biosystems). Control mRNAs (luciferase, α Factor and β lactamase) were all from Promega. *In vitro* translation (IVT) reactions were carried out using nuclease-free rabbit reticulocyte lysates (Promega) with 0.5–1.0 µg mRNA. Mycolactone was diluted in 5% (w/v) BSA in nuclease-free water before addition, controls contained BSA alone. Where used, canine microsomal membrane (CPMM) preparations (Promega, or prepared in house [58]; a kind gift of Prof Barnhard Dobberstein [University of Heidelberg]) or semi-permeabilised RAW264.7 cell extracts freshly prepared as described in [59] were added to a final concentration of 10%. Samples were incubated for 30 min at 30°C. For protease protection assays, samples were diluted in 1∶5 in 20 mM TrisCl pH 8.0, 10 mM CaCl_2_ and split into 3 aliquots: to the control sample buffer alone was added to a final volume of 50 µl, the second aliquot contained 20 µg/ml Proteinase K and the third 20 µg/ml Proteinase K and 0.1%Triton-x-100. Samples were incubated for 1 hr at 4°C, then reactions were stopped by addition of 5 mM PMSF and boiling sample load buffer. For glycosylation assays of tail-anchored proteins and PPaF, ES1 was pre-incubated with in-house CPMM for one hour before the addition to other components. Mycolactone-containing CPMM were used immediately. Membranes were recovered as described [31] by centrifugation through 750 mM sucrose, 500 mM KOAc, 5 mM Mg(OAc)_2_, 50 mM HEPES-KOH (pH 7.9) at 100,000×*g* for 10 mins. The membrane pellet was resuspended in 100 mM sucrose, 100 mM KOAc, 5 mM Mg(OAc)_2_, 50 mM HEPES-KOH (pH 7.9), 1 mM DTT and treated with 250 µg/ml RNaseA at 37°C for 10 mins to remove any residual peptidyl-tRNA species. Samples were separated by SDS-PAGE. Luciferase, α Factor, β lactamase, Sec61β and Cyt-B5 were detected by labelling with ^35^S methionine; TNFα was detected by SDS-PAGE (15% acrylamide) and Western blotting.

### DNA amplification

Total RNA was extracted from cell lysates or digitonin permeabilised fractions using the RNeasy kit (Qiagen) and quantified by Nanodrop. One-step qRT-PCR gene expression assays (Life Technologies) were carried out on either an Applied Biosystems 7900 (Life Technologies) or on a Stratagene Mx3005P (Stratagene). For relative gene expression the ΔΔCt method was used. For absolute quantitation, full-length murine cDNAs were prepared and used to form standard curves. XBP-1 splicing was investigated by RT-PCR of total RNA followed by Pst1 digestion as described [60].

### Immunochemistry

Secreted TNF was detected in culture supernatants by ELISA. For Western blots, cells were lysed directly in gel sample buffer then sonicated or permeabilised with digitonin. Proteins were separated by SDS-PAGE (12.5% acrylamide) followed by conventional blotting. Where quantitation was performed, pixel density was assessed using ImageJ analysis of non-saturated images and data were normalized to an appropriate loading control (GAPDH)

### Metabolic labelling

Metabolic labelling was performed as previously described [5]. Met/Cys-starved cells were stimulated as described above for 2 hrs before the addition of 0.37MBq Tran35S-Label for a further 2 hr at 37°C. They were then either lysed in 20 mM TrisCl, pH 7.4, 0.5M NaCl, 1% Triton-X-100. 1× protease inhibitor cocktail) or separated into cytosolic and membrane fractions as described above. Samples were separated by SDS-PAGE, exposed to a phosphorimager screen and analysed using a Personal FX imager (BioRad).

### Accession numbers

α factor (PPaF), β-actin: P60710, BiP: P20029, Cox-2: Q05769, Cytochrome b5: P00167, EIF2α: Q6ZWX6, GAPDH: P16858, Glucosidase 1: Q80UM7, IL6: P08505, β lactamase: Q9L5C7, Luciferase: P08659,: P01149, PABP1: P29341, PERK: Q9Z2B5, TNF (murine): P06804, TNF (human): P01375, Sec61β: P60468, XBP1: Q6ZWX6.

## Supporting Information

Figure S1 **Synthetic mycolactone prevents production of proinflammatory proteins in RAW264.7 cells by a post-transcriptional mechanism and is not cytotoxic under the conditions used.** A. RAW264.7 cells were incubated for 1 hr +/− various concentrations of mycolactone (as indicated), 0.5 µg/ml Actinomycin D (Act D) or 0.0125% DMSO then stimulated or not with LPS for 4 hr. A. Cell viability as assessed by MTT assay, expressed as a percentage of control cells (mean±SEM, n = 3). B. Primary human monocyte-derived macrophages or RAW264.7 cells were treated for 1 hr with natural mycolactone A/B (MYC), 2 µg/ml ActD, 10 µg/ml cycloheximide (CHX) or 0.001% EtOH then stimulated with LPS for 2 hrs. TNF in supernatants was quantified by ELISA and normalised to production without inhibitors; Mean±SEM of 4 independent experiments (separate donors for primary cells). C. RAW264.7 cells were incubated +/−125 ng/ml mycolactone for 1 hr then stimulated with LPS for 4 hr before harvesting for polysome profiling. Left panel: Supernatant TNF levels at time of harvest as determined by ELISA. Right panel: total RNA from a portion of cell lysate was used as a template for qRT-PCR relative gene expression assays for TNF mRNA.
(TIF)

Figure S2 **TNF secretion is not affected by short term exposure to puromycin and homoharringtonine.** A. RAW264.7 cells were incubated +/−125 ng/ml mycolactone for 1 hr then stimulated with LPS for 4 hr then incubated with puromycin (PURO) or homoharringtonine (HH) for 3 mins as described in the legend of Fig. 2. Immediately before lysing, supernatants were harvested and assayed for TNF by ELISA (Mean±SEM of triplicate values as percentage of control).
(TIF)

Figure S3 **Mycolactone inhibits the secretion of most cytokines, chemokines and other inflammatory mediators.** RAW264.7 cells were incubated for 1 hr +/−125 ng/ml mycolactone then stimulated or not with LPS overnight (16 hrs) and supernatants were used undiluted to probe the Mouse Cytokine Array, Panel A. A. Cytokines detected by the array (R&D systems (http://www.rndsystems.com/product_detail_objectname_mousecytokinearraypanela.aspx). The orange and yellow highlights indicate proteins that were detected with either high (normalised intensity >1.0) and low (normalised intensity <1.0) abundance, respectively B. Pixel intensity was determined using ImageJ software and normalised according to the positive control signals. Values represent the mean of duplicate spots ± range. The asterisk (*) represents proteins that are produced constitutively by RAW264.7 cells C. Raw data for the arrays; reference spots (used to orient the array and to normalise between arrays) are boxed. Proteins produced by LPS stimulated cells are indicated for reference only; orange; strong intensity, yellow, weaker intensity. Note that expression is relative between treatments and differences in intensity between proteins are not necessarily quantitative. D. RAW264.7 cells were incubated +/−125 ng/ml mycolactone for 1 hr then stimulated with LPS for overnight. TNF and MIP-1α levels were measured by ELISA.
(TIF)

Information S1 **Extended experimental procedures.** Contains a full list of reagents, primers and antibodies as well as detailed protocols including those for polysome preparation, digitonin permeabilisation and ConA precipitation.
(PDF)
