# Supplementary figures and images for: The Pathogenic Mechanism of the Mycobacterium ulcerans Virulence Factor, Mycolactone, Depends on Blockade of Protein Translocation into the ER
Source: PLoS Pathog. 2014 Apr 3;10(4):e1004061. doi: 10.1371/journal.ppat.1004061 (PMC3974873; doi:10.1371/journal.ppat.1004061)

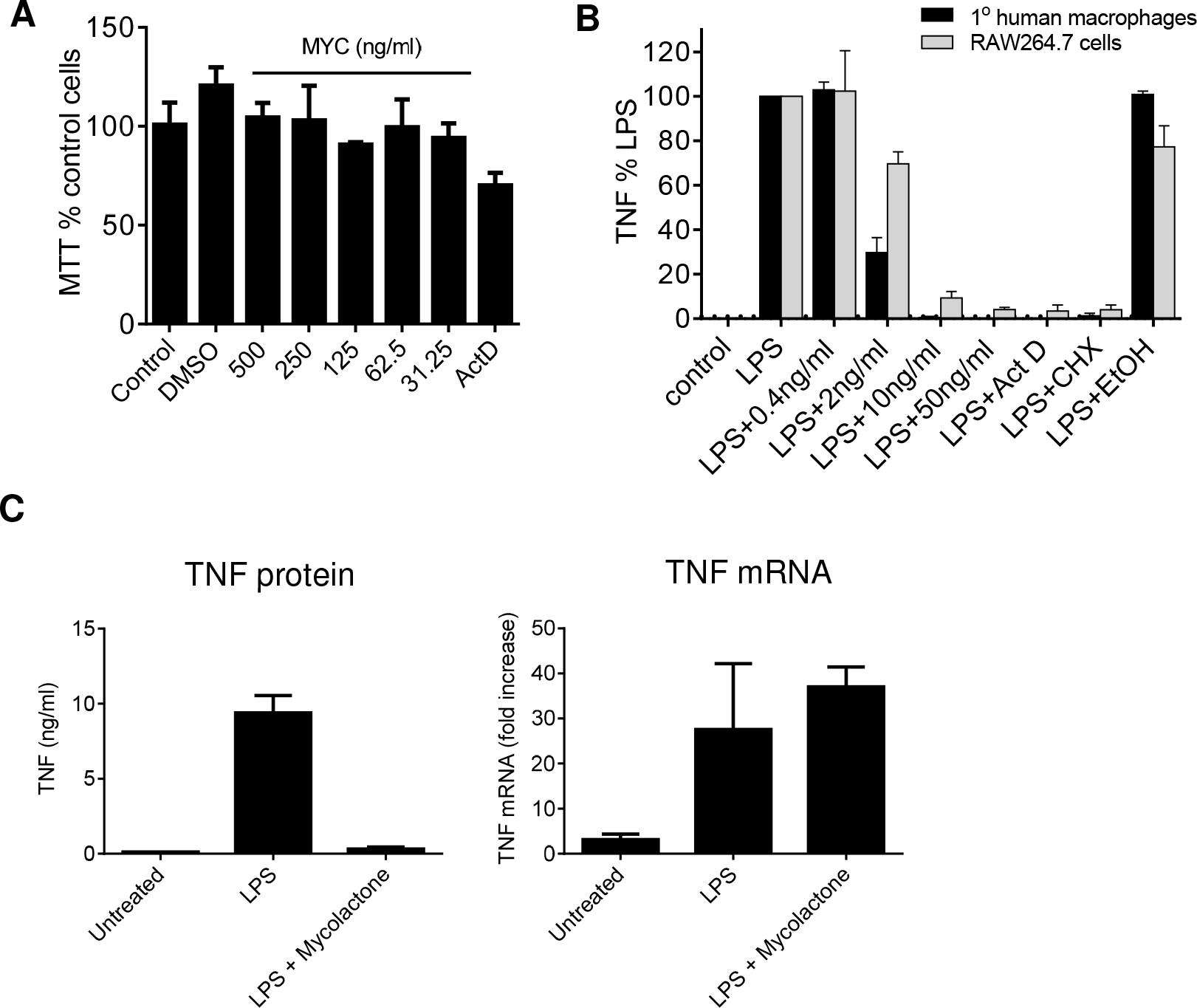

Supplement: Figure S1 — Synthetic mycolactone prevents production of proinflammatory proteins in RAW264.7 cells by a post-transcriptional mechanism and is not cytotoxic under the conditions used. A. RAW264.7 cells were incubated for 1 hr +/− various concentrations of mycolactone (as indicated), 0.5 µg/ml Actinomycin D (Act D) or 0.0125% DMSO then stimulated or not with LPS for 4 hr. A. Cell viability as assessed by MTT assay, expressed as a percentage of control cells (mean±SEM, n = 3). B. Primary human monocyte-derived macrophages or RAW264.7 cells were treated for 1 hr with natural mycolactone A/B (MYC), 2 µg/ml ActD, 10 µg/ml cycloheximide (CHX) or 0.001% EtOH then stimulated with LPS for 2 hrs. TNF in supernatants was quantified by ELISA and normalised to production without inhibitors; Mean±SEM of 4 independent experiments (separate donors for primary cells). C. RAW264.7 cells were incubated +/−125 ng/ml mycolactone for 1 hr then stimulated with LPS for 4 hr before harvesting for polysome profiling. Left panel: Supernatant TNF levels at time of harvest as determined by ELISA. Right panel: total RNA from a portion of cell lysate was used as a template for qRT-PCR relative gene expression assays for TNF mRNA. (TIF) [file ppat.1004061.s001.tif]

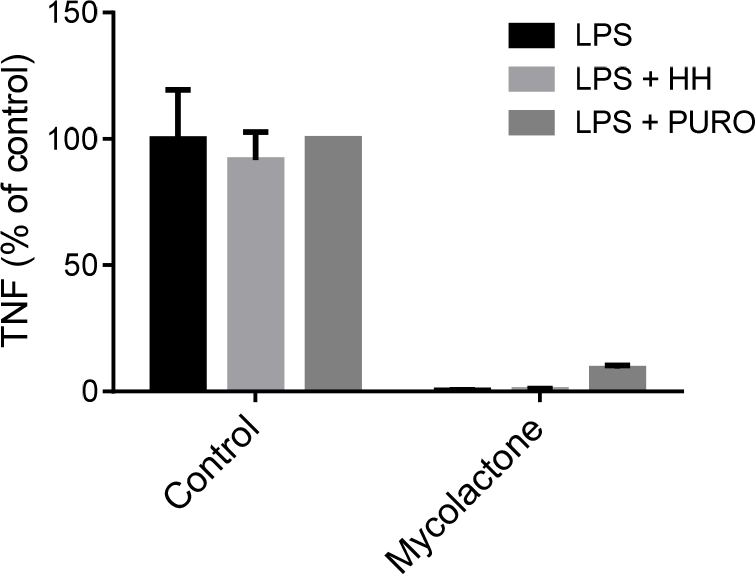

Supplement: Figure S2 — TNF secretion is not affected by short term exposure to puromycin and homoharringtonine. A. RAW264.7 cells were incubated +/−125 ng/ml mycolactone for 1 hr then stimulated with LPS for 4 hr then incubated with puromycin (PURO) or homoharringtonine (HH) for 3 mins as described in the legend of Fig. 2. Immediately before lysing, supernatants were harvested and assayed for TNF by ELISA (Mean±SEM of triplicate values as percentage of control). (TIF) [file ppat.1004061.s002.tif]
