# Supplementary material for: The Pathogenic Mechanism of the Mycobacterium ulcerans Virulence Factor, Mycolactone, Depends on Blockade of Protein Translocation into the ER
Source: PLoS Pathog. 2014 Apr 3;10(4):e1004061. doi: 10.1371/journal.ppat.1004061 (PMC3974873; doi:10.1371/journal.ppat.1004061)
Supplement: Information S1 — Extended experimental procedures. Contains a full list of reagents, primers and antibodies as well as detailed protocols including those for polysome preparation, digitonin permeabilisation and ConA precipitation. (PDF) [file ppat.1004061.s004.pdf]

Information S1 - Extended Experimental Procedures

Reagents

All chemicals were obtained from Sigma Aldrich unless otherwise stated.

Mycolactone

The mycolactone used in this study was mycolactone A/B in a 3:2 mixture of the stereoisomers. With the exception of Fig S1C, all of the experiments in this work utilised synthetic mycolactone A/B [1] provided by Dr Kishi in ethyl acetate. This was dried under N_2 and resuspended in sterile tissue culture grade DMSO and stored in aliquots at -80℃ in the dark. Figure S1C utilised natural mycolactone A/B, extracted from *M. ulcerans* 1615 and provided by Prof Small, was stored in ethanol at -20℃ in the dark. The purity of both preparations was verified by LC/MS (not shown). We could find no difference in the activity or stability of mycolactone in the different solvents but storage in DMSO has considerable advantages in terms of handling and conservation of material.

Additional cell culture

Primary human monocytes were purified from plateletphoresis residues as previously described [2] and differentiated into monocyte-derived macrophages in 100ng/ml M-CSF (Peprotech) for 4 days. Primary human macrophages were maintained in RPMI (PAA) supplemented with 5% FBS (Biosera) and used immediately. Primary human dermal microvascular endothelial cells were purchased from Lonza and were cultured according to their instructions in EGM-2MV (Lonza) at passage <7. Both HeLa and L929 fibroblasts were maintained in high-glucose DMEM supplemented with 10% FBS.

Antibodies

ELISAs used either commercial kits according to the manufacturer's instructions (murine TNF and MIP-1α: Ready-SET-Go!® Kit, eBioscience) or paired antibodies (human TNF and

IL-6: BD Biosciences) as described [2]. Anti Cox-2 (SC1745), anti TNF (SC1351) and anti glucosidase I (SC374006) antibodies were from Santa Cruz Laboratories. Anti-BiP, anti-phospho-PERK, anti-eIF2α and eIF2α were from Cell Signalling Technology. Anti GAPDH was from Ambion. All secondary antibodies were from Life Technologies except anti-goat-HRP (Dako).

Cytokine array

For the cytokine array, LPS-stimulation of RAW264.7 cells proceeded overnight and culture supernatants were clarified by centrifugation for 5min at 5000xg. These supernatants were then used undiluted to probe the R&D Systems Mouse Cytokine Array, Panel A according to the manufacturer's instructions. Pixel density was quantified using ImageJ analysis of non-saturated images with background subtraction and normalisation according to reference spots.

Primers

Off-the-shelf gene expression assays used were: TNF (Hs_00174128_m1, Mm_ 0043258_m1), IL-6 (Mm_0046190_m1), Cox-2 (Mm_00478374_m1) and GAPDH (4352934). Real-time one-step qRT-PCR was carried out with either One-Step RT-PCR Master Mix (Applied Biosystems) on the 7900 or Brilliant III Ultrafast qRT-PCR Master Mix (Stratagene) on the Mx3005P. The primers used for conventional *XBP1* RT-PCR amplification were 5'-AAACAGAGTAGCAGCGCAGACTGC-3' and 5'-TCCTTCTGGGTAGACCTCTGGGAG-3'.

Polysome preparation

For isolation of polysomal RNA, 1-2 x 10^7 cells were incubated with 10µg/ml cycloheximide (CHX) for 10min at 37°C and 5% CO_2. For translation inhibition experiments, either puromycin (100µg/ml) or homoharringtonine (5µM) (Enzo Life Sciences) were added 3min prior to addition of CHX. Cells were placed on ice and washed with ice cold PBS containing

10μg/ml CHX and harvested by scraping into PBS/CHX and spun at 450x*g* for 5min at 4℃. The cell pellet was resuspended in 500μl lysis buffer (15mM TrisCl pH 7.5, 300mM sodium chloride, 15mM magnesium chloride, 10mg/m^1 heparin, 100μg/ml CHX and 1% (v/v) Triton-x-100). Lysates were clarified by centrifugation at 21000x*g* for 1min at 4℃ and supernatants snap frozen in liquid nitrogen and stored at -80℃. No difference was observed between profiles derived from frozen and freshly prepared lysates. To separate polysomes, samples (400μl) were layered onto a 10-50% sucrose gradient in lysis buffer and centrifuged in an SW40Ti rotor (Beckmann Coulter) at 38,000 rpm for 2hr. Gradients were fractionated using a FoxyR1 collection system (Teledyne ISCO) and absorbance was followed at 254nm. Fractions (1ml) were collected into tubes containing 3ml 7.7M guanidium chloride (Melford), then 4ml 100% ethanol was added. Samples were mixed and incubated overnight at -80℃, then RNA was purified as described by Johannes and Sarnow [3]. For Northern blotting, extracted RNA was separated on 1% agarose/formaldehyde gels and transferred onto Hybond N+ membranes. Blots were probed in Ultrahyb solution (GE Healthcare) with ^{32}P-labelled cDNA probes washed and exposed to a phosphorimager screen (BioRad). Full-length coding-region cDNA probes for actin and PABP were provided by Anne Willis (University of Leicester). Murine TNF, IL-6 and Cox-2 probes were prepared by amplification and cloning of full-length coding-region cDNAs from LPS-stimulated RAW264.7 cells.

Digitonin permeabilisation

Membrane bound and cytosolic RNA were separated by digitonin permeabilisation using the method of Stephens *et al* [4] with minor alterations. Briefly, 10^6 RAW264.7 cells were exposed to mycolactone and stimulated as described above for 4hrs. Monolayers were placed on ice and washed twice with ice-cold PBS then permeabilised in 300μl Permeabilisation Buffer (110mM potassium acetate, 25mM HEPES pH7.2, 2.5mM magnesium acetate, 1mM EGTA, 0.03% digitonin, 1mM DTT, 1x protease inhibitor cocktail and 40U ml^{-1} RNasin [Promega]) for 10min at 4℃ with rocking. The supernatant, (representing the cytosolic fraction) was removed and clarified by centrifugation at 7500x*g*

for 10min at 4°C. Cells were washed with ice-cold Wash Buffer (110mM potassium acetate, 25mM HEPES pH7.2, 2.5mM magnesium acetate, 1mM EGTA, 0.004% digitonin, 1mM DTT and 1x protease inhibitor cocktail). Membrane proteins were solubilised in 300µl Lysis Buffer (400mM potassium acetate, 25mM HEPES pH7.2, 15mM magnesium acetate, 1% (v/v) NP-40, 0.5% (w/v) sodium deoxycholate, 1mM DTT, 1 x protease inhibitor cocktail and 40U ml^{-1} RNasin) for 10min at 4°C with rocking. The supernatant (representing the membrane fraction) was removed and clarified by centrifugation at 7500x*g* for 10min at 4°C.

ConA precipitation

ConA agarose (Vector Labs) was washed three times in 20mM TrisCl, pH7.4, 1M NaCl, 5mM $MgCl_2$, 5mM $MnCl_2$, 5mM $CaCl_2$ and resuspended in the same buffer to give a 50:50 slurry then 20µL was added to each lysate or supernatant. Samples were incubated for 1hr on ice then centrifuged at 1000*g* for 1min. Pellets were washed three times in lysis buffer then resuspended in sample loading buffer for separation by SDS-PAGE.

References

1. Song F, Fidanze S, Benowitz AB, Kishi Y (2002) Total synthesis of the mycolactones. Org Lett 4: 647-650.

2. Simmonds RE, Lali FV, Smallie T, Small PL, Foxwell BM (2009) Mycolactone inhibits monocyte cytokine production by a posttranscriptional mechanism. J Immunol 182: 2194-2202.

3. Johannes G, Sarnow P (1998) Cap-independent polysomal association of natural mRNAs encoding c-myc, BiP, and eIF4G conferred by internal ribosome entry sites. RNA 4: 1500-1513.

4. Stephens SB, Dodd RD, Lerner RS, Pyhtila BM, Nicchitta CV (2008) Analysis of mRNA partitioning between the cytosol and endoplasmic reticulum compartments of mammalian cells. Methods Mol Biol 419: 197-214.
